# Two-Dimensional Sagittal-Plane Gait Evaluation and Similarity Analysis in Parkinson’s Disease Under ON and OFF Conditions: A Pilot Study

**DOI:** 10.3390/brainsci16040385

**Published:** 2026-03-31

**Authors:** Jocabed Mendoza-Martínez, Fiacro Jiménez-Ponce, Karla Nayelli Silva-Garcés, Sergio Rodrigo Méndez García, Adolfo Angel Casarez Duran, Christopher René Torres-SanMiguel

**Affiliations:** 1 Instituto Politécnico Nacional, Escuela Superior de Ingeniería Mecánica y Eléctrica, Unidad Zacatenco, Sección de Estudios de Posgrado e Investigación, Ciudad de México 07738, Mexico; jmendozam1903@alumno.ipn.mx (J.M.-M.);; 2 Centro de Neurociencias, Hospital Ángeles Pedregal, Ciudad de México 10700, Mexico

**Keywords:** biomechanics, Parkinson’s disease, sagittal-plane kinematics, freezing gait, videogrammetry

## Abstract

**Background/Objectives:** Freezing of gait (FoG) is a disabling motor manifestation of Parkinson’s disease (PD) associated with impaired neural control of locomotion and increased gait variability. Quantitative characterization of gait kinematics may provide biomechanical insight into FoG-related instability, particularly under different dopaminergic states. **Methods:** This pilot study evaluated sagittal-plane knee kinematics in healthy individuals (*n* = 27) and patients with PD. (*n* = 8) under OFF and ON dopaminergic medication conditions using two-dimensional videogrammetry (Kinovea^®^). Knee flexion–extension trajectories were time-normalized to 0–100% of the gait cycle, and group ensemble profiles (mean ± SD) were computed. **Results**: Phase-specific range of motion (ROM), within-subject variability, and interlimb coordination were quantified. Interlimb coordination was assessed using Pearson’s correlation coefficients (r) and cross-correlation lag analysis computed per subject and summarized statistically across groups. Compared with healthy participants, PD patients in the OFF state exhibited significantly reduced knee ROM during stance and swing (*p* < 0.05), accompanied by increased kinematic variability and disrupted temporal coordination. Interlimb correlation was significantly lower in PD OFF compared to healthy gait groups (*p* = 0.010), with larger temporal lags, indicating impaired bilateral synchronization. Following medication intake (ON state), knee excursion increased and interlimb coordination partially improved; however, correlation values and timing symmetry did not fully normalize to healthy levels. **Conclusions:** These findings demonstrate that sagittal-plane knee kinematics and interlimb coordination metrics derived from low-cost 2D videogrammetry are sensitive to the dopaminergic state and reveal persistent neuromotor deficits in PD. The proposed framework provides an interpretable and accessible approach for characterizing gait organization in Parkinson’s disease and supports future integration with clinical assessment and longitudinal monitoring.

## 1. Introduction

Human gait is a locomotion process in which the human body, in an upright position, moves forward, with its weight supported by both legs. It is a forward-swinging movement in preparation for the next foothold [1]. Human gait is a complex phenomenon that considers many variables and areas of knowledge to understand its movements, sequence, energy requirements, reaction forces between the feet and the ground, and the different muscles involved [2]. The upright position of a human is unstable, so the body requires a greater neuronal and motor effort, which is learned during childhood. Factors that modify and vary each person’s gait pattern include sex, height, BMI, and age. Other secondary factors that alter gait pattern are the speed of progression, mood, footwear, clothing, and terrain type. For A. E. Minetti [3], the mechanics of walking uphill and downhill reveal the connection between the limitations of the human locomotor system and its metabolic demands [4,5].

A clinical approach could define the gait cycle during sagittal-plane analysis. The sagittal plane is relevant for studying neural control of gait, as forward progression and rhythmic limb flexion–extension are predominantly governed by sagittal-plane joint motion [6,7,8,9]. Sagittal balance is described as the principle of the overall support moment, like the algebraic summation of sagittal-plane lower-extremity joint extensor moment of force during gait [10,11]. This hypothesis is based on the concept that the central nervous system controls individual joint moments of force and equilibrium. It is an act of the entire lower extremity that works as a single unit regarding gravity support.

There is also a conditioning that involves the “automatic step.” The automatization of walking is developed during infancy [12]. As people age, brain damage and loss of muscular tone can affect gait [13]. Older people’s gait is also described as cautious, emphasizing maximum stability and safety, and as a slowed version of walking. Significant changes occur after the age of 60.

Additionally, degenerative diseases increase the number of people with gait pathology, which directly impacts their quality of life. Two persistent, prevalent disorders involved are osteoarthritis and Parkinson’s disease (PD) [14,15].

PD is a neurological progressive degenerative disorder that involves symptoms such as tremors, rigidity, bradykinesia, speech disorders, loss of automatic movements, and impaired posture and balance [16,17]. PD arises due to the degeneration of dopaminergic neurons in the substantia nigra pars compacta (SNpc) [18]. Alongside motor symptoms, non-motor manifestations are also prominent, which include depression, anxiety, cognitive deterioration, and symptoms related to autonomic nervous system failures [19]. These symptoms are broadly classified into motor, non-motor, and dysautonomia symptoms [20]. Treatment of PD consists of a combination of medications, such as levodopa/carbidopa, dopaminergic agonists, adenosine antagonists, amantadine, and anticholinergics, as well as surgical treatments, such as deep brain stimulation, that help control movement and improve quality of life [21]. In addition to pharmacological therapy, deep-brain stimulation (DBS) has emerged as an important treatment option for patients with advanced Parkinson’s disease. DBS involves the surgical implantation of electrodes in specific brain regions, most commonly the subthalamic nucleus (STN) or the globus pallidus interna (GPi), that deliver continuous electrical stimulation to modulate abnormal neural activity in the basal ganglia circuitry. This intervention has been shown to significantly improve several motor symptoms of Parkinson’s disease, including tremor, rigidity, and bradykinesia. However, its effects on gait and postural stability remain variable and are still undergoing investigation. Understanding how locomotor patterns change under different therapeutic conditions, including dopaminergic medication states and DBS modulation, is, therefore, essential for improving clinical assessment and rehabilitation strategies for individuals with Parkinson’s disease.

A combination of rigidity and bradykinesia during the gait cycle, also called freezing of gait (FoG) [22], is a clinical condition in which the down limbs freeze and suddenly stop. This condition is observed in the advanced stage of PD [23,24].

Researchers have been studying the gait cycle in PD using various methods to measure walking, including analytical methods (e.g., accelerometers) [25,26,27]. Two-dimensional videogrammetry provides a quantitative window into neural abnormalities associated with PD by capturing how motor commands are translated into movements. In particular, kinematic features describing movement amplitude and temporal modulation are prominent in Parkinsonian gait and are associated with impaired motor flexibility. This method calculates movements, velocity, and acceleration in different studies, such as falls. However, this method is also used for gait analysis for upper and lower limbs [28]. Alterations in this plane directly reflect deficits in movement scaling and timing, the core manifestation of impaired neural locomotor control in PD. Moreover, sagittal-plane analysis allows robust, reproducible assessment using two-dimensional videogrammetry, making it suitable for clinical and translational applications.

This study proposes a descriptive method for obtaining the main variables from subjects at various stages of the gait cycle. Although gait impairment in Parkinson’s disease has been extensively investigated using wearable sensors, inertial systems, and 3D motion capture technologies, limited attention has been given to phase-normalized sagittal-plane ensemble analysis using low-cost, marker-based two-dimensional videogrammetry under controlled ON/OFF dopaminergic conditions. To our knowledge, no prior study has systematically combined phase-normalized knee kinematics, interlimb correlation analysis, and cross-correlation lag metrics derived from accessible 2D videogrammetry to quantify medication-dependent neuromotor alterations in Parkinson’s disease. This study addresses this methodological gap by providing a reproducible, low-cost framework that captures phase-specific kinematic organization and bilateral coordination changes associated with dopaminergic modulation. This approach aims to bridge laboratory-based biomechanics with clinically feasible gait assessment tools. The goal is to gather information to support a study of PD performance using 2D videogrammetry in sagittal mode, focusing on knee flexo-extension.

## 2. Materials and Methods

An observational transversal study was conducted. The selection criteria were healthy subjects without gait comorbidities (without orthopedic surgery) and patients with PD with gait disturbance but walking condition (age 60 and above, diagnosis of PD according to the Movement Disorder Society (MDS) criteria, the ability to walk independently or with minimal assistance, Hoehn–Yahr stage III, levodopa/carbidopa responsiveness in stable doses, without metal deterioration, and with deep brain stimulation (DBS) treatment). Due to the heterogeneity of the participants, the scoping review method [29,30] was used to collect information and characterize the samples based on demographic data (sex, age, and anthropometric characteristics). Video analysis was performed using Kinovea^®^ (Kinovea-Version 2023.1.2). After the recording phase, the parameters were analyzed using statistical methods.

**Figure 1:**
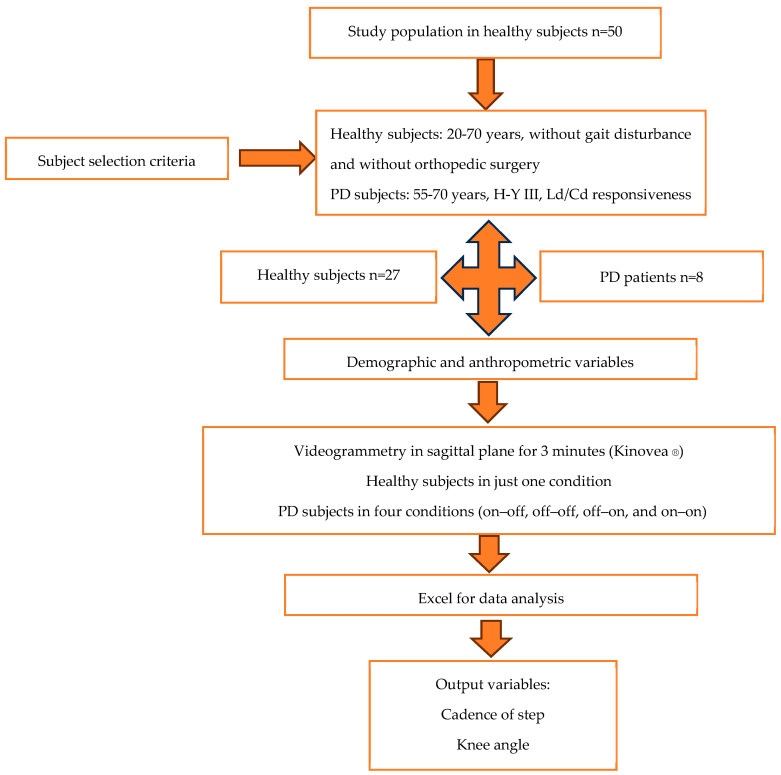
Diagram for gait analysis according to scoping review. PD participants were evaluated in four conditions: OFF medication/OFF DBS, ON medication/OFF DBS, OFF medication/ON DBS, and ON medication/ON DBS. H-Y = Hoehn and Yahr scale. Ld/Cd = levodopa/carbidopa.

This methodology was used in healthy participants and people with PD. The following diagram (Figure 1) describes the complete method for obtaining all criteria and parameters. Initially, 50 healthy volunteers were screened. Twenty-three participants were excluded due to incomplete gait cycles (*n* = 23), tracking loss during videogrammetric analysis (*n* = 10), or failure to meet predefined quality control thresholds for continuous marker visibility and phase segmentation (*n* = 13). The final healthy control sample included 27 participants. For Parkinson’s participants, 20 participants (*n* = 20) were selected; 7 were excluded for not presenting freezing of gait (*n* = 7) and 5 for failure to meet predefined quality control thresholds for continuous marker visibility (*n* = 5).

The data selection criteria were delineated to obtain data from non-standardized human bodies to include all different body types. Every subject needed to be healthy and free of visible walking comorbidities. The age range of 17 to 36 years was covered. Twenty-seven volunteers were selected. For subjects with Parkinson’s, the sample size was set at 8 participants. Table 1 presents the average demographic data for healthy subjects and patients with Parkinson’s disease.

Measurements of body sections were taken to obtain the first descriptive statistics. The data were obtained from the left and right upper arm (LUA and RUA), left and right lower arm (LLA and RLA), left and right upper leg (LUL and RUL), and left and right lower leg (LLL and RLL). With these data, we aimed to establish a database that can be augmented with information to improve the average criteria score and create a corridor of FoG for Parkinson’s patients.

Given the pilot nature of this study, a post hoc power analysis was performed using G*Power 3.1 to estimate the achieved statistical power for primary ROM comparisons. For the observed effect sizes (Cohen’s d = 0.69–1.98), the statistical power ranged between 0.72 and 0.95 at α = 0.05, indicating moderate to high sensitivity despite the limited PD sample size (*n* = 8).

This study included a cohort of healthy subjects and patients diagnosed with Parkinson’s disease (PD). All participants were able to walk independently without assistive devices. For patients with PD, gait data were collected under ON and OFF dopaminergic medication conditions, in accordance with standard clinical practice. Healthy subjects were recorded under a single condition and served as the control reference group. All procedures were conducted per ethical standards for human research, and informed consent was obtained from all participants before data collection.

### 2.1. Experimental Design

#### Marker Placement and Experimental Setup

**Figure 2:**
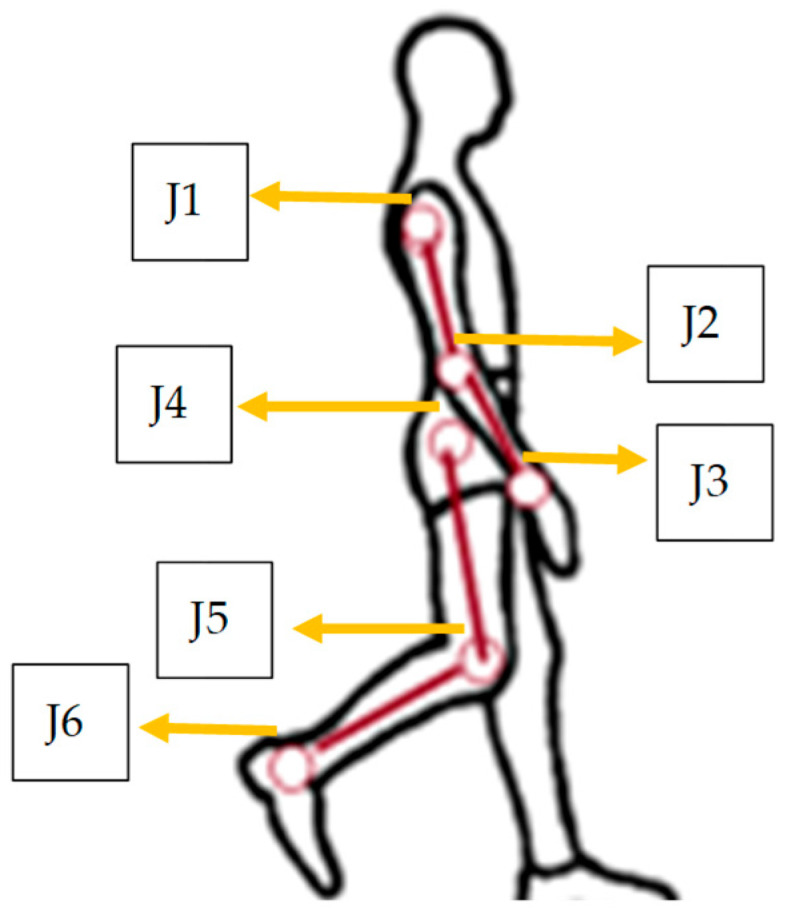
Diagram of joint marker relation with body parts. Placement of reflective markers used for sagittal-plane videogrammetry. Joint landmarks were defined as follows: J1—shoulder joint; J2—elbow joint; J3—wrist joint; J4—hip joint (greater trochanter); J5—knee joint (lateral femoral condyle); J6—ankle joint (lateral malleolus). These markers were used to track limb kinematics during the gait cycle.

A special black cloth suit was used to fix the reflection markers. These markers were placed on the principal joints, with 6 for the upper limbs and 6 for the lower limbs. Figure 2 shows the markers’ locations in every joint, with six for each side at the shoulder (J1), the elbow (J2), the wrist (J3), the hip (J4), the knee (J5), and the ankle (J6).

### 2.2. Scenario Setup

**Figure 3:**
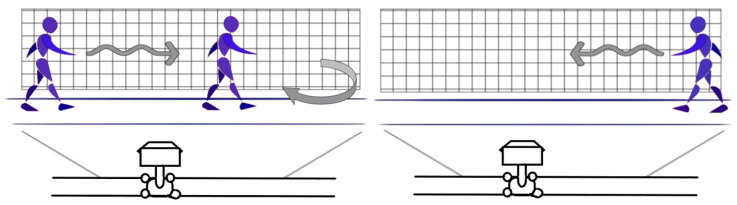
Gait analysis scenario. The arrows indicaste the back and forth walking path within the camera view.

A specific scenario was created to perform the gait cycle. A series of prior instructions was given to the participants. One of the requirements was to wear comfortable clothes. Healthy people performed the test with comfortable shoes, and patients with Parkinson’s performed the test with their usual shoes. The gait cycle scenario (Figure 3) was defined by a 5.5 m long, 60 cm wide corridor, using a sagittal camera (model: GoPro 13 made by GoPro, Inc., which is based in San Mateo, CA, USA). A distance of 1.85 m from the center of the gait scenario to the camera was settled. The scenario was divided into a 10 × 10 cm grid. A calibration grid with known dimensions (10 × 10 cm spacing) was physically placed within the gait corridor during recording. This grid was subsequently referenced within the Kinovea^®^ software to establish spatial scaling and convert pixel measurements into real-world distances.

### 2.3. Videogrammetry

To analyze the activity, a sagittal recording of the walk was captured with a GoPro 13 camera, which supports high-speed recording in MP4 and WVA formats. The camera was mounted on a 2 m rail to follow the person. The scenario had 3 principal lights to reflect the markers: a frontal light, a lateral light, and a light mounted on the rail. The participants’ gait was analyzed using the Kinovea^®^ software, focusing on the knee joint. Key reference points included the hip initiation point (greater trochanter), the knee marker (lateral condyle), and the ankle marker (lateral malleolus), as illustrated in Figure 2. The recording length was 3 min.

### 2.4. Clinical Conditions to Assess the Subject

For healthy participants, the clinical conditions were focused on no gait disturbance and no surgical or orthopedic assistance. PD participants were evaluated in four conditions: OFF medication/OFF DBS, ON medication/OFF DBS, OFF medication/ON DBS, and ON medication/ON DBS.

On medication referred to the usual effect of dopaminergic medication. Off medication referred to assessments after withdrawal of dopaminergic medication for at least 12 h. DBS could be turned off on-site. The effect was instantaneous. The videogrammetry processes in the PD group were the same as in healthy subjects.

### 2.5. Ethical Approval

This study was approved by the Comité de Investigación y de Ética en Investigación de Operadora de Hospitales Ángeles S.A. de C.V. (approval code: HAP 2771; approval date: 3 April 2025). All procedures were conducted in accordance with the Declaration of Helsinki and institutional guidelines for human research. Written informed consent was obtained from all participants before data acquisition.

### 2.6. Equations

Once the data were obtained from the Kinovea^®^ program, the information was classified, and the flexo-extension of the left and right legs was quantified. Once this information was filtered, we performed statistical analysis and filtering. For the statistical part, we proceeded to obtain averages, which allowed us to perform a similarity analysis of the gait corridors obtained from videogrammetry. This analysis consisted of a correlation and a cross-correlation; the choice was motivated by the need to characterize both structural similarity and temporal coordination in the sagittal plane. Correlation [31] is a statistical measure that quantifies the strength and direction of a relationship between two variables. It is commonly measured using Pearson’s correlation coefficient (r), which ranges from −1 to 1:

r = 1 → Perfect positive correlation (as one increases, the other increases).
r = −1 → Perfect negative correlation (as one increases, the other decreases).
r = 0 → No correlation (no linear relationship).

Mathematically, the Pearson correlation coefficient between two signals *x* and *y* is

(1) $$r=\frac{\sum (x_{i}-\bar{x})(y_{i}-\bar{y})}{{\sum (x_{i}-\bar{x})}^{2}{\sum (y_{i}-\bar{y})}^{2}}$$

Cross-correlation [32] extends the concept of correlation by analyzing how much one signal resembles another when shifted in time. It is used in signal processing to detect time-lagged relationships between signals.

For two continuous-time signals *x*(*t*) and *y*(*t*), the cross-correlation function is

(2) $$R_{xy}(\tau )=\int_{-\infty }^{\infty }x(t)y(t+\tau )dt$$

For discrete signals, it is

(3) $$R_{xy}[k]=\sum_{n=-\infty }^{\infty }x[n]y[n+k]$$

where *τ* (or *k*) represents the time lag between signals, which can be used for gait analysis. In this case, we used discrete signals.

#### Statistical Normalization of Gait Data

A two-level statistical normalization strategy was applied to address both temporal and amplitude-related variability inherent to human gait to enable a meaningful comparison of gait kinematics between healthy subjects and patients with PD. Statistical significance and effect magnitude were jointly considered to ensure robust interpretation of phase-specific gait differences.

Raw knee angle trajectories were filtered using a fourth-order zero-lag Butterworth low-pass filter with a cutoff frequency of 6 Hz, selected based on the typical frequency content of human gait kinematics. The sampling frequency was 120 Hz (GoPro frame rate). Zero-phase filtering was implemented using forward–reverse digital filtering to prevent phase distortion. Following filtering, gait cycles were segmented and temporally normalized to 0–100% using real-time scaling (1501 ms = 100%).

Normality was assessed using the Shapiro–Wilk test for all variables, and paired differences were used to assess conformity to normality. A significance threshold of p≥0.05 indicated approximate normality, guiding the selection of parametric or non-parametric tests.

For primary hypothesis testing, comparisons between stance and swing were performed using paired *t*-tests or Wilcoxon signed-rank tests. For between-group comparisons (healthy vs. PD), the data were evaluated using Welch’s *t*-test for normally distributed data or the Mann–Whitney U test due to violations of normality and unequal sample sizes. A non-parametric test was used for primary interference. A two-tailed significance level of α=0.05 was adopted.

Effect size quantification for complement *p*-values and the magnitude of observed differences was using Cohen’s *dz*, which was computed for paired comparisons:

(4) $$dz=\frac{\bar{d}}{s_{d}}$$

where $\bar{d}$ represents the mean paired difference, and $s_{d}$ represents its standard deviation.

Cohen’s d was calculated for independent group comparisons:

(5) $$d=\frac{\bar{x_{1}}-\bar{x_{2}}}{s_{p}}$$

where $s_{p}$ denotes the pooled standard deviation. The effect sizes were interpreted as small = 0.2; medium = 0.5; and large = ≥0.8.

## 3. Results

### 3.1. Videogrammetry Analysis of Healthy Subjects

The final sample comprised 27 healthy volunteers. The parameters analyzed were from sagittal-plane gait, where each individual’s gait pattern was measured to obtain an average across these samples. Table 2 shows the variability in the angles about knee flexion–extension and the mean obtained for each participant. The variability in the samples was due to the participants, as not all were within a specific percentile.

Figure 3 shows the record and how to obtain individual data on lower-limb behavior, using three points to generate this graph. These were the markers placed at the hip, knee, and ankle joints, respectively, with a central marker at the knee joint.

**Figure 4:**
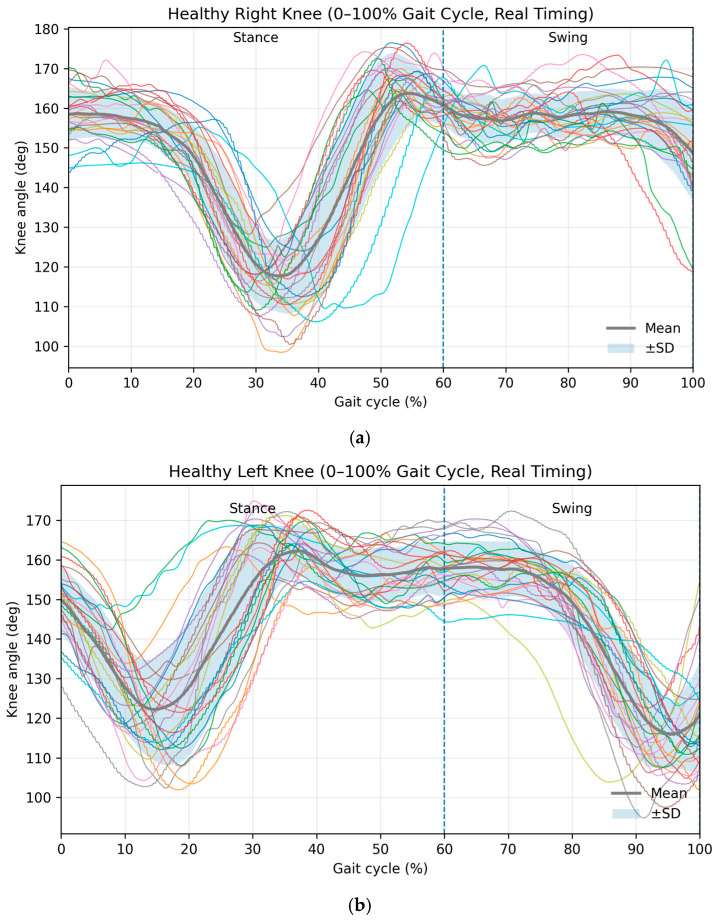
Ensemble knee flexion–extension trajectories of healthy participants for the (**a**) right and (**b**) left limbs, normalized to 0–100% of the gait cycle using real temporal scaling. Individual subject waveforms are shown with the group mean (Colores lines) and the average of behavioral waveforms (solid black line) and ±1 standard deviation (shaded region). The vertical dashed line at 60% denotes the stance-to-swing transition (toe-off). High waveform overlap and consistent phase structure indicate stable interlimb coordination and low kinematic variability in neurologically healthy gait.

As shown in Table 2, healthy participants were measured on both sides (P1–P27), and a paired design was used. The normality of right–left differences was assessed using the Shapiro–Wilk test. The mean knee angle and ROM differences did not violate normality (*p* > 0.05), so paired *t*-tests were appropriate. Variability (within-subject SD) differences violated normality (*p* = 0.024); therefore, the Wilcoxon signed-rank test was used as the primary inferential test for that feature. Across all features, no statistically significant right–left differences were observed, and effect sizes were small, supporting expected sagittal-plane symmetry in healthy gait. In Figure 4, the left leg shows greater constancy, whereas the right leg shows variability in its maximum extension points, as reflected in the angles produced during gait analysis. Healthy participants exhibited highly consistent sagittal knee kinematic profiles across the gait cycle for both limbs. During the stance phase (0–60%), a progressive flexion pattern followed by extension was observed, corresponding to weight acceptance and propulsion mechanics. The swing phase (60–100%) demonstrated increased knee flexion, facilitating limb clearance and forward advancement. Low dispersion among individual trajectories and narrow standard deviation envelopes indicated strong motor control consistency and minimal inter-subject variability. Right- and left-limb waveforms displayed comparable amplitudes and temporal structure, reflecting preserved bilateral coordination during healthy locomotion.

### 3.2. Videogrammetry Analysis from Subjects with Parkinson’s

**Figure 5:**
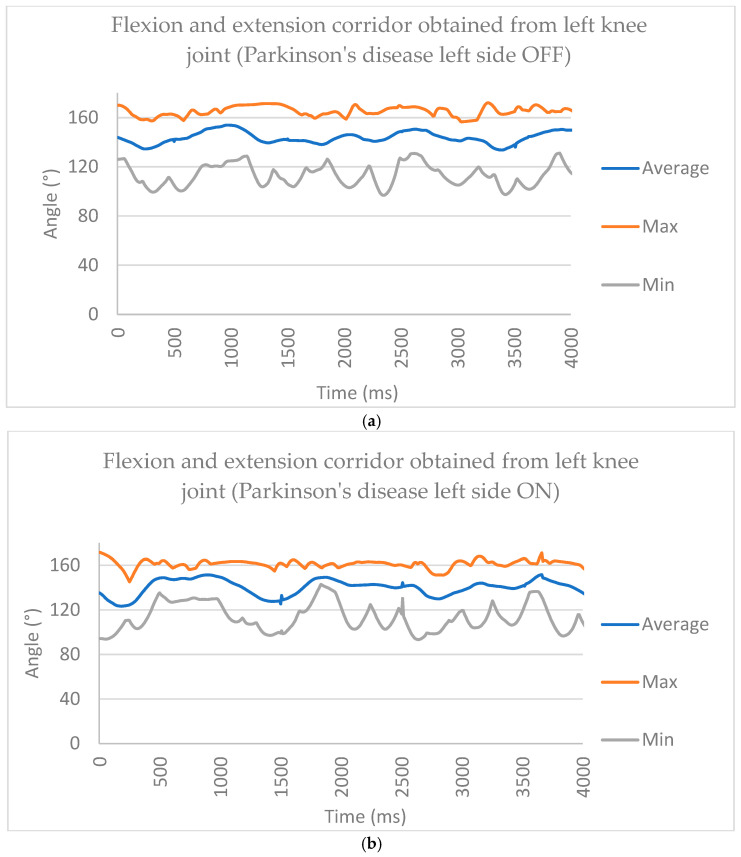

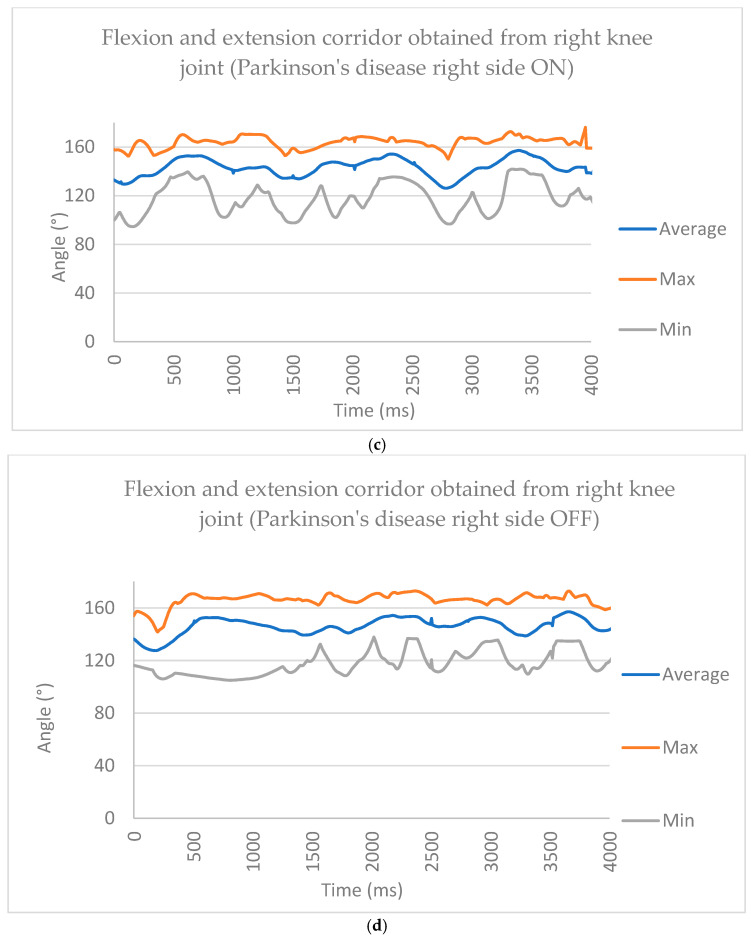
For people with Parkinson’s disease, a corridor for patients with no medication. The behavior was erratic because of the disease. (**a**) Parkinson’s disease left side OFF ad (**b**) Parkinson’s disease right side OFF. (**c**) Parkinson’s disease left side ON and (**d**) Parkinson’s disease right side ON.

For people with PD, gait analysis was performed in two stages: during OFF gait, which referred to the person not taking their medication (hence, the Parkinson’s pathology became more evident as they approached their next dose), and during the ON gait, which referred to when the patient had already taken their dose of medication and could perform the gait within 30 min to 1 h. In Figure 5, we observe the gait pattern during OFF gait, wherein walking was erratic, and each gait cycle took slightly longer to complete. For ON, the cycle of gait had a better performance.

**Figure 6:**
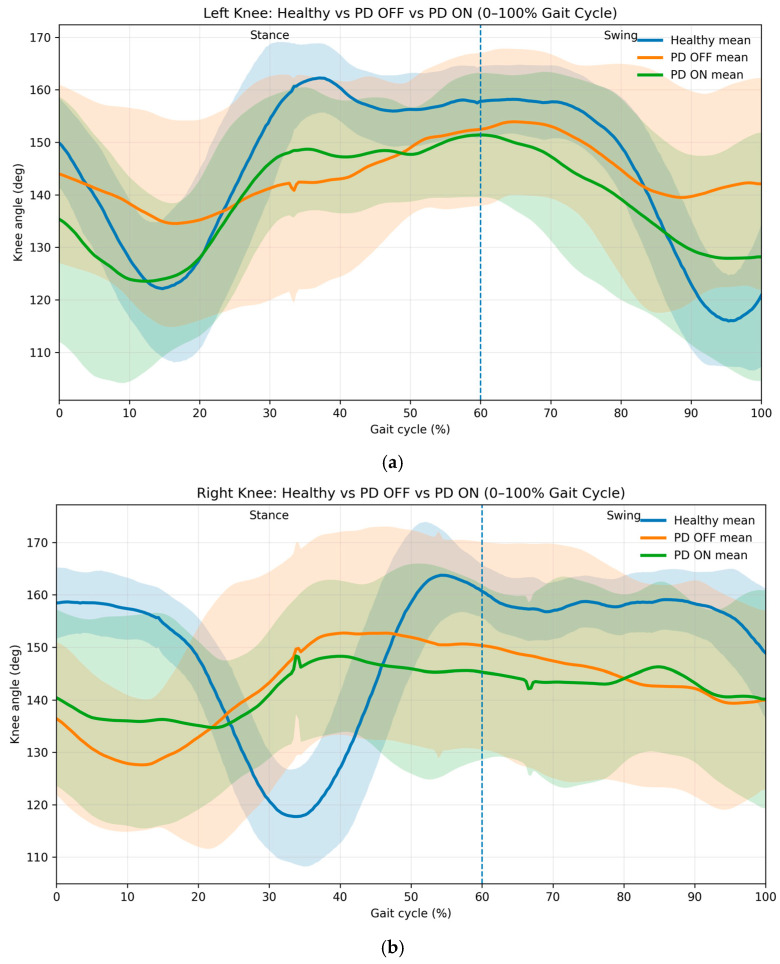
Mean sagittal-plane knee flexion–extension across the gait cycle for healthy vs. Parkinson’s disease patients. (**a**) Left knee kinematic profile showing attenuated flexion–extension amplitude and increased variability in Parkinson’s disease during the OFF state, with partial normalization following medication intake. (**b**) Right knee kinematic profile demonstrating asymmetric impairment in Parkinson’s disease OFF and incomplete recovery during the ON state relative to healthy controls. The shaded areas show the main behavorail for every state, the blue represents Healthy Participants, the orange one represents Parkinson disease without assistance of medicine and DBS, and the green one represents Parkinson disease with assistance of medicine and DBS. The dashed lines represent the gait cycle for normalized gait at 60% for stance phase and 40% of swing phase.

To create the corridor in the gait analysis in Parkinson’s disease (PD), we used the same methodology as that shown in Figure 5, where we used the average, min, and max functions to create corridor graphics for the OFF and ON scenarios. Their behavior can be observed in Figure 6. The mean sagittal-plane knee flexion–extension trajectories across the gait cycle for healthy participants, Parkinson’s disease patients in the OFF medication state, and Parkinson’s disease patients in the ON medication state are shown. All curves were normalized to 0–100% of the gait cycle using real temporal scaling (1501 ms = 100%). Shaded areas represent ±1 standard deviation across participants. The vertical dashed line indicates toe-off (60%), separating stance and swing phases. Parkinson’s disease OFF demonstrates reduced range of motion and increased variability compared with healthy gait, whereas ON medication partially restores movement amplitude and temporal coordination.

Phase-normalized knee kinematics revealed marked alterations in Parkinson’s disease relative to healthy gait. In the OFF-medication state, patients exhibited reduced knee excursion throughout both stance and swing phases, accompanied by substantially greater inter-subject variability. Following medication intake (ON state), knee flexion amplitude increased and temporal coordination improved, particularly during stance-to-swing transition; however, trajectories did not fully converge with healthy patterns. According to Table 3, we can observe the statistical description.

Phase-specific analysis revealed pronounced alterations in knee kinematics in patients with Parkinson’s disease. Compared with healthy subjects, PD participants exhibited significantly reduced range of motion during stance and swing phases for both limbs (*p* < 0.05), accompanied by marked increases in movement variability, particularly during transition and swing-related intervals. Effect sizes were consistently moderate to large (Cohen’s d = 0.66–1.98), indicating strong biomechanical deviations rather than minor statistical differences. The largest impairments occurred during late stance and early swing, corresponding to gait phases associated with dynamic weight transfer and limb advancement. These findings suggest disrupted neuromotor coordination in PD, consistent with impaired basal ganglia modulation of rhythmic locomotion.

Both limbs demonstrated persistent deviations during swing, indicating residual neuromotor deficits despite pharmacological intervention.

A post hoc statistical power analysis was conducted based on the observed effect sizes for the primary kinematic outcomes to assess the adequacy of the sample size. Using the group sizes included in this study (27 healthy participants and eight patients with Parkinson’s disease) and a significance level of α = 0.05, the achieved statistical power ranged from 0.36 to 1.00, depending on the magnitude of the effect size. Comparisons with large effect sizes (Cohen’s d ≈ 1.29–1.98) showed high statistical power (0.87–1.00), whereas comparisons with moderate effect sizes (Cohen’s d ≈ 0.66–0.69) showed lower statistical power (0.36–0.38). These results reflect the exploratory nature of this pilot study and the limited availability of clinically eligible Parkinson’s disease participants.

### 3.3. Correlation and Cross-Correlation Analysis

**Figure 7:**
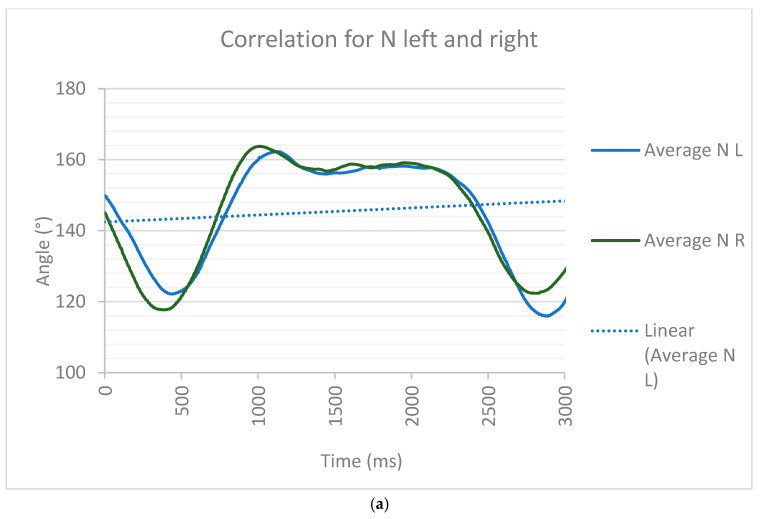

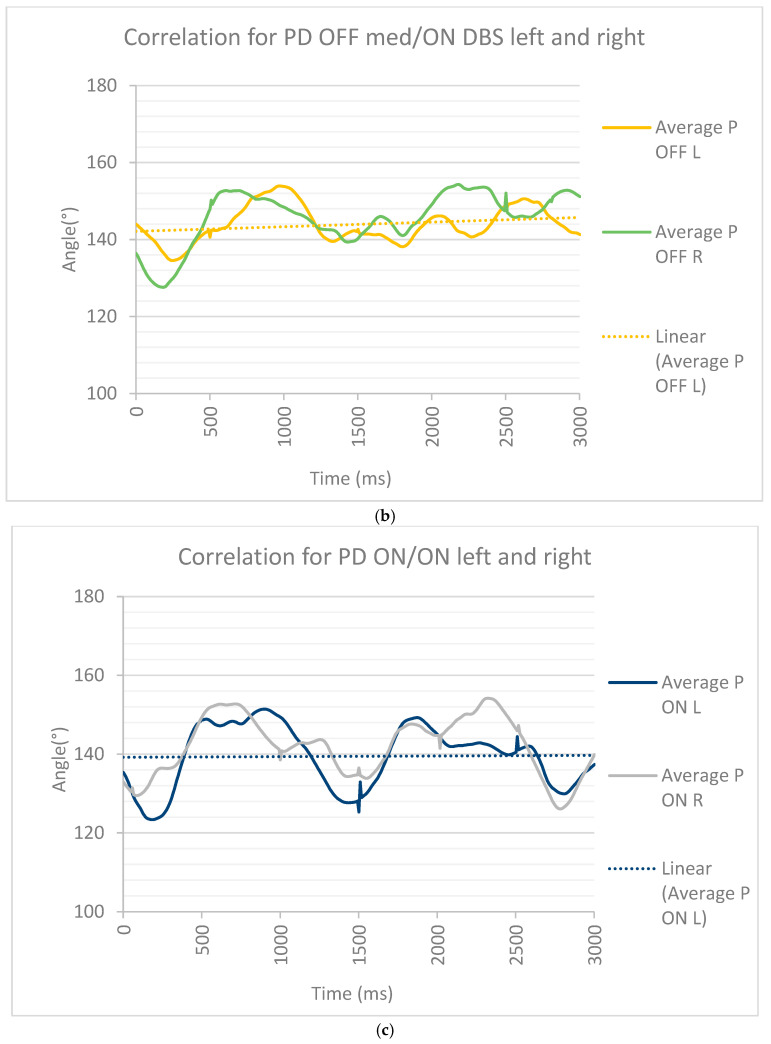
Correlation analysis of sagittal-plane knee flexion–extension trajectories. (**a**) Healthy participants showed high bilateral similarity (mean r = 0.92; range: 0.88–0.96). (**b**) Parkinson’s disease participants exhibited reduced coordination in the OFF medication state (mean r = 0.61; range: 0.42–0.78). (**c**) With partial improvement following medication intake (ON state: mean r = 0.73; range: 0.55–0.85).

Here, we analyzed the correlation and cross-correlation of the corridors obtained from healthy and Parkinson’s patients. For this study, we used the correlation coefficient for healthy patients (N) to assess the similarity of the average gait corridors shown in Figure 6. Figure 7a shows that the analysis of the correlation between the left (L) and right (R) legs using their averages was very similar, so applying Equation (1), we obtained an r = 0.9176 that had a positive correlation close to 1. Correlation and cross-correlation analyses were performed separately for the health control and PD groups. For each group, a representative reference waveform (Figure 7) was obtained by averaging time-normalized sagittal-plane knee flexion–extension trajectories across subjects. The Pearson correlation coefficients were computed between individual subject waveforms and subsequently applied to assess temporal alignment and identify potential phase shifts relative to the reference waveform. These analyses were used to characterize within-group organization and coordination rather than to directly compare left and right limbs. This shows that the walking corridor for healthy people can serve as a reference to differentiate it from a Parkinson’s corridor.

Figure 7 shows the differences between Parkinson’s corridors and those of healthy people. The analysis of results from videogrammetry 2D found significant differences in the correlation between healthy and PD patients with ON/OFF medication/DBS. In Figure 7a, a specific pattern of walking is shown in healthy subjects. In the swing phase, the difference in the knee flexo-extension was about 40° in a duration of 500 ms for healthy people. In PD OFF, medication/ON DBS patients, the knee flexion–extension difference was around 20°, with a duration of 250 ms. The same analysis between healthy and PD ON/ON patients showed a flexo-extension of about 25° and a duration of 350 ms. PD OFF/OFF could not be evaluated because the patient was unable to stand up.

In the stance phase, for healthy people, the angle variation was minimal (around 5°), and the duration was around 1100 ms. In PD OFF medication/ON DBS, the variation in the angle was around 14°, and the duration was at least 3000 ms. For PD ON/ON, the variation in the angle was around 9°, and the duration was at least 625 ms.

Healthy participants exhibited strong similarity between left and right knee flexion–extension trajectories, with a mean Pearson correlation coefficient of r = 0.92 (range: 0.88–0.96). In contrast, Parkinson’s disease participants demonstrated reduced interlimb coordination in the OFF medication condition, with a mean correlation coefficient of r = 0.61 (range: 0.42–0.78). After dopaminergic medication intake (ON condition), the correlation values partially improved to r = 0.73 (range: 0.55–0.85), although they remained lower than those observed in healthy participants.

The average time-normalized knee flexion–extension trajectories of the left and right limbs are shown for (a) healthy subjects, (b) Parkinson’s disease patients in the OFF medication state, and (c) Parkinson’s disease patients in the ON state. High waveform overlap in healthy subjects indicated strong bilateral coordination, whereas reduced alignment in PD OFF reflected impaired interlimb synchronization. Partial restoration of coordination was observed in the ON condition. Linear trend lines illustrate overall kinematic coupling across the gait cycle.

Using the correlation between these data, Table 4 shows the value of r with respect to the Parkinson’s statistical R comparison.

**Figure 8:**
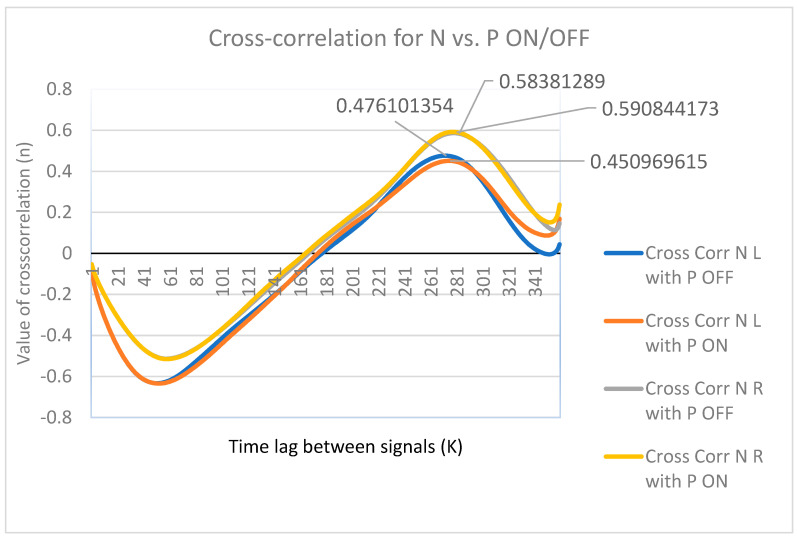
People with Parkinson’s disease in the corridor using cross-correlation with healthy participants.

Interlimb coordination was quantified for each subject using Pearson’s correlation and cross-correlation lag between left and right knee kinematic trajectories. Healthy participants exhibited moderate correlations with minimal temporal offsets, reflecting physiological anti-phase limb coordination. In contrast, Parkinson’s disease patients in the OFF medication state demonstrated either excessive synchronization (r > 0.8) or near-complete loss of coupling (r ≈ 0), accompanied by large timing shifts, indicating disrupted neural rhythm generation. Following medication intake, coordination partially improved in several subjects; however, abnormal synchronization and residual timing asymmetries persisted, suggesting incomplete restoration of sensorimotor integration. We performed cross-correlation to discretize flexo-extension behavior as a signal to establish how similar walking behavior was across both scenarios. Interlimb coordination differed significantly between groups, with a marked disruption observed in the PD OFF condition compared to healthy participants (*p* = 0.010; Table 5).Once this cross-correlation was carried out, as shown in Figure 8, the similarity value was obtained from the y-axis, which indicated that the R/L for the OFF state of R = 0.46± 0.025 and the R/L for the ON state of R = 0.585± 0.007, indicating that it presented similarities of 46% and 58%, respectively.

## 4. Discussion

This study investigated sagittal-plane knee kinematics and interlimb coordination in healthy individuals and patients with Parkinson’s disease using two-dimensional videogrammetry. By explicitly quantifying movement amplitude, variability, and temporal coordination across the gait cycle, the analysis provides biomechanically interpretable markers of altered locomotor control in PD. It clarifies the effects of dopaminergic medication on gait organization.

The findings align with previous research indicating that PD patients exhibit slower walking speeds, reduced stride length, and increased gait variability [33]. These changes are often linked to the degeneration of dopaminergic neurons in the substantia nigra, which leads to motor control deficits. This study highlights that gait disturbances in PD are not uniform but can vary significantly by disease stage and the presence of other motor or non-motor symptoms, such as freezing of gait (FOG). Previous studies have reported that individuals with PD often exhibit diminished movement amplitude across lower-limb joints, reflecting dysfunction in basal ganglia circuits responsible for regulating motor timing and amplitude. In this study, the reduction in knee excursion during both stance and swing phases is consistent with these reports and likely reflects impaired neuromotor control of rhythmic locomotion.

This study confirms earlier findings that PD is associated with significant alterations in gait dynamics, including reduced stride length, increased variability, and bradykinetic movements (Balestrino & Schapira, 2020) [16]. Specifically, the range of motion at the knee joint during the swing phase in PD patients in the OFF medication state was markedly reduced (~20° over 250 ms) compared with healthy participants (~40° over 500 ms). These discrepancies were slightly reduced under ON medication/ON DBS conditions (~25° over 350 ms), but remained distinct from healthy norms, indicating partial, rather than complete, restoration of motor function [34].

Previous studies of gait analysis using technological methods, such as videogrammetry, could be integrated into clinical practice. Andressa Fiori Bortoli et al. [35] presented a compilation of 20 participants: 10 older adults who were apparently healthy and 10 with PD. The study evaluated the range of motion during water walking, which served as a point of comparison with the results. This established a range of motion of 70°, which is similar to Figure 7, where the range is around 55°, but not to Figure 8, where the range of motion is around 43°. This may be because Figure 8 shows patients in the OFF (without medication) state, and Figure 7 shows patients in the ON (with medication) state. This range of motion follows a line and involves walking on the ground.

This study used correlation (r = 0.9176) and cross-correlation analyses to quantify the symmetry and consistency of knee flexo-extension during gait. While healthy subjects demonstrated high interlimb coordination, PD patients exhibited substantial asymmetry and temporal disorganization, particularly in the OFF state. These findings echo the prior literature emphasizing gait asymmetry and the progressive deterioration of neuromotor coordination in PD (Nussbaum & Ellis, 2003; Weiss et al., 2020) [17,22].

This research also supports the feasibility of using gait corridor analysis as a diagnostic and monitoring tool. The cross-correlation values (~0.46 for OFF and ~0.58 for ON states) suggest that even in altered gait patterns, there is a detectable individual “gait fingerprint” that could be tracked over time. This opens avenues for integrating videogrammetry with real-time monitoring systems using wearable sensors and machine learning algorithms to predict motor fluctuations [36].

A key contribution of this work is the explicit, per-subject quantification of interlimb coordination. In healthy participants, left and right knee trajectories exhibited high correlation coefficients with minimal temporal lag, reflecting a stable bilateral coordination typical of physiological anti-phase gait.

In contrast, PD patients in the OFF state showed significantly reduced interlimb correlation and increased timing lags, indicating disrupted bilateral synchronization. This disruption reflects impaired neural coupling between limb oscillators rather than simple asymmetry. Following medication intake, correlation coefficients increased, and timing lags decreased in several subjects, demonstrating partial restoration of interlimb coordination. However, substantial inter-subject variability persisted, emphasizing heterogeneity in medication response.

Correlation and cross-correlation analyses were used to characterize within-group coordination structure, not to infer causality or diagnose freezing of gait. This distinction addresses methodological ambiguity and strengthens interpretability.

An important contribution of this study is the demonstration that meaningful biomechanical information can be obtained using two-dimensional videogrammetry and open-source analysis tools such as Kinovea^®^. While three-dimensional motion capture systems remain the gold standard for gait analysis, they require specialized laboratory environments and substantial financial resources. In contrast, the approach presented here offers a more accessible means of capturing phase-dependent kinematic features and interlimb coordination patterns in clinical populations. Such low-cost methods may facilitate broader implementation of quantitative gait analysis in clinical and translational research settings.

Several limitations should be considered when interpreting these results. First, the size of the Parkinson’s disease participant sample was relatively small, reflecting the difficulty of recruiting clinically eligible individuals who met the study criteria. Second, there was a notable age difference between the control and Parkinson’s disease groups, which may independently influence gait characteristics. Although phase-normalized analysis and effect-size reporting were used to mitigate potential confounding factors, future studies should aim to include age-matched control groups to isolate disease-specific biomechanical alterations better. Finally, the present analysis focused on sagittal-plane knee kinematics; future work should incorporate additional joints and three-dimensional measurements to provide a more comprehensive characterization of Parkinsonian gait.

Despite these limitations, the present findings demonstrate that sagittal-plane knee kinematic analysis derived from two-dimensional videogrammetry can capture clinically relevant differences between healthy and Parkinsonian gait patterns. The approach provides a practical and accessible framework for investigating locomotor coordination and movement variability in Parkinson’s disease and may serve as a foundation for future studies exploring gait impairments and rehabilitation strategies in this population. This level of precision is unmatched and extremely valuable in both clinical gait labs and high-performance sports science [37].

Future studies should incorporate age-matched controls, walking speed normalization, and standardized clinical scales (e.g., UPDRS and FoG-Q). Longitudinal designs and integration with wearable sensors or machine learning models may further enhance sensitivity to disease progression and motor fluctuations.

Overall, this study’s findings contribute to the understanding of gait alterations in Parkinson’s disease by highlighting phase-specific changes in knee kinematics and disruptions in interlimb coordination. These results reinforce the importance of biomechanical analysis in characterizing locomotor deficits and support the potential application of accessible videogrammetric methods for studying gait abnormalities in neurological disorders.

## 5. Conclusions

This study demonstrates the practical utility of 2D videogrammetry using Kinovea^®^ software for analyzing gait abnormalities in individuals with Parkinson’s disease (PD), particularly in the context of freezing of gait (FoG). This research highlights significant differences in gait parameters between healthy individuals and PD patients across various clinical conditions (ON/OFF medication and DBS) using sagittal-plane analysis of knee flexion–extension.

The findings confirm that patients with PD exhibited distinct patterns of gait irregularity—such as reduced range of motion, increased variability, and asymmetry—especially in OFF states. These patterns were objectively quantified using correlation and cross-correlation analyses, providing a reproducible, statistically supported method for distinguishing pathological gait from standard patterns.

This study underscores the potential of low-cost, accessible tools such as Kinovea for clinical and research applications. While Kinovea lacks the precision and three-dimensional capability of high-end systems such as Vicon, it offers sufficient accuracy and usability for many diagnostic and monitoring purposes, especially in resource-limited settings. Kinovea’s simplicity, affordability, and portability make it an excellent option for preliminary assessments and longitudinal monitoring of gait disturbances in PD.

However, due to the small sample size (These data can be found within Supplementary Material), further research is needed to validate these preliminary results. Future work should focus on expanding the sample population to include diverse age groups and comorbid conditions, and on integrating wearable sensors and machine learning algorithms for real-time gait analysis and feedback.

Although this study was limited by sample size and age differences between groups, the findings support the utility of knee joint kinematics and interlimb coordination metrics as quantitative markers of Parkinsonian gait dysfunction. Future work should incorporate age-matched cohorts, standardized clinical scales, and longitudinal monitoring to validate these measures further and explore their potential integration with machine learning models for disease staging and therapeutic assessment.

## Figures and Tables

**Table 1:** The average demographic data included subjects with Parkinson’s disease. Abbreviations: BMI = body mass index; LUA = left upper arm; RUA = right upper arm; LLA = left lower arm; RLA = right lower arm; LUL = left upper leg; RUL = right upper leg; LLL = left lower leg; RLL = right lower leg; W = women; M = male.

Demographic Data	Healthy Subject	Standard Deviation	PD Subject	Standard Deviation
Subjects	27		8	
Gender	M = 18, W = 9		M = 7, W = 1	
Age	26.48	±4.38	62.25	±0.5
Weight (kg)	74.27	±16.03	75.5	±8.89
Height (cm)	167.9	±8.43	168.8	±4.83
BMI	26.17	±4.32	26.69	±2.25
LUA	33.3	±2.35	31.25	±2.06
RUA	33.44	±2.25	31.375	±2.06
LLA	26.22	±1.98	24.75	±1.26
RLA	26.46	±1.96	24.62	±1.25
LUL	46.72	±3.34	45.5	±2.38
RUL	46.7	±3.40	45.5	±2.38
LLL	42.31	±3.75	40.62	±2.14
RLL	42.3	±3.88	40.5	±2.08

**Table 2:** Healthy subjects: right vs. left sagittal-plane knee features.

Feature	Left (Mean ± SD)	Right (Mean ± SD)	Normality of Diff. (Shapiro *p*)	Primary Test	*p*-Value	Effect Size (Cohen’s d_z)
Mean knee angle (deg)	146.92 ± (between-subject SD)	148.23 ± (between-subject SD)	0.112	Paired *t*-test	0.161	0.26
ROM (deg)	63.81 ± (between-subject SD)	63.25 ± (between-subject SD)	0.199	Paired *t*-test	0.665	−0.08
Within-subject SD (deg)	17.06 ± (between-subject SD)	17.61 ± (between-subject SD)	0.024	Wilcoxon	0.080	0.27

**Table 3:** Phase-specific knee kinematic metrics in healthy and PD subjects.

Side	Phase	Metric	Healthy (Mean ± SD)	PD (Mean ± SD)	*p*-Value	Effect Size (d)
Left	Stance	ROM	49.13 ± 13.71	40.85 ± 7.96	0.016	0.69
Left	Swing	ROM	49.31 ± 9.88	33.03 ± 15.59	0.001	1.32
Left	Stance	Variability	15.75 ± 4.97	12.87 ± 3.00	0.023	0.66
Left	Swing	Variability	17.71 ± 4.36	11.24 ± 6.00	<0.001	1.29
Right	Stance	ROM	55.04 ± 6.10	39.52 ± 10.16	<0.001	1.98
Right	Swing	ROM	17.49 ± 9.47	30.95 ± 17.20	0.015	1.05
Right	Stance	Variability	17.69 ± 2.02	13.01 ± 3.88	<0.001	1.64
Right	Swing	Variability	4.25 ± 2.55	10.25 ± 6.45	0.006	1.37

**Table 4:** Effect size (Cohen’s d) and achieved power for phase-specific knee range-of-motion and variability measures.

Outcome	Cohen’s d	Achieved Power
Left stance ROM	0.69	0.38
Left swing ROM	1.32	0.89
Left stance variability	0.66	0.36
Left swing variability	1.29	0.87
Right stance ROM	1.98	1.00
Right swing ROM	1.05	0.72

**Table 5:** Statistical summary of interlimb correlation (r).

Comparison	*p*-Value	Interpretation
Healthy vs. PD OFF	0.010	Significant disruption
Healthy vs. PD ON	0.157	Partial normalization
PD OFF vs. PD ON	0.699	High inter-subject variability

## Data Availability

The data presented in this study are openly available in Google drive at https://drive.google.com/drive/folders/1bFivId7K_DWQZ8tCzKKKgVHSubFFAFim (accessed on 29 January 2026).

## Supplementary Materials

The following supporting information can be downloaded at https://drive.google.com/drive/folders/1bFivId7K_DWQZ8tCzKKKgVHSubFFAFim (accessed on 29 January 2026).
